# Implicit visual sensitivity towards slim versus overweight bodies modulates motor resonance in the primary motor cortex: A tDCS study

**DOI:** 10.3758/s13415-020-00850-0

**Published:** 2020-12-01

**Authors:** Stergios Makris, Valentina Cazzato

**Affiliations:** 1 Department of Psychology, Edge Hill University, Ormskirk, Lancashire L39 4QP UK; 2 Arts and Wellbeing Research Centre, Edge Hill University, Ormskirk, UK; 3 School of Psychology, Faculty of Health, Liverpool John Moores University, Tom Reilly Building, Byrom Street, Liverpool, L3 3AF UK

**Keywords:** Action observation, Primary motor cortex, tDCS, Motor resonance, Anti-fat attitudes

## Abstract

Motor resonance (MR) can be influenced by individual differences and similarity in the physical appearance between the actor and observer. Recently, we reported that action simulation is modulated by an implicit visual sensitivity towards normal-weight compared with overweight bodies. Furthermore, recent research has suggested the existence of an action observation network responsible for MR, with limited evidence whether the primary motor cortex (M1) is part of this. We expanded our previous findings with regards to the role of an implicit normal-weight-body preference in the MR mechanism. At the same time, we tested the functional relevance of M1 to MR, by using a transcranial direct current stimulation (tDCS) protocol. Seventeen normal-weight and 17 overweight participants were asked to observe normal-weight or overweight actors reaching and grasping a light or heavy cube, and then, at the end of each video-clip to indicate the correct cube weight. Before the task, all participants received 15 min of sham or cathodal tDCS over the left M1. Measures of anti-fat attitudes were also collected. During sham tDCS, all participants were better in simulating the actions performed by normal-weight compared with overweight models. Surprisingly, cathodal tDCS selectively improved the ability in the overweight group to simulate actions performed by the overweight models. This effect was not associated with scores of fat phobic attitudes or implicit anti-fat bias. Our findings are discussed in the context of relevance of M1 to MR and its social modulation by anti-fat attitudes.

## Introduction

Mounting research evidence indicates that the observation of one person performing an action can automatically activate a network of cortical brain regions in the observer associated with action execution. This phenomenon is known as “motor resonance” (MR), and it is considered a critical element of one’s ability to anticipate forthcoming actions and make predictions about their outcome (Aglioti, Cesari, Romani, & Urgesi, 2008; Fadiga, Fogassi, Pavesi, & Rizzolatti, 1995; Urgesi et al., 2010). Furthermore, it has been recently shown that MR can be modulated by one’s experience with the observed action (Abreu et al., 2012; Buccino et al., 2004), whereas physical and psychological similarities between the actor and observer also can influence the level of action simulation and anticipatory representation of other’s actions (Avenanti, Sirigu, & Aglioti, 2010; Azevedo, Macaluso, Viola, Sani, & Aglioti, 2014; Cazzato & Makris, 2019; Obhi, Hogeveen, Giacomin, & Jordan, 2014).

The ability to perceive and simulate others’ actions is deemed as critical in social interactions, and it is reciprocally linked to the empathic ability to understand someone’s intentions and emotions (Gapinski, Schwartz, & Brownell, 2006). For example, recent studies on pain perception have indicated that both physical similarity and group membership can influence the empathic resonant neural responses to others’ pain (Avenanti et al., 2010; Azevedo et al., 2013). More recently, we have shown that MR can be modulated by differences in the body weight between the actor and observer and that explicit negative attitudes toward overweight models can influence our ability to accurately simulate and predict the outcome of their actions (Cazzato & Makris, 2019). This is an interesting finding indicating that not only previous experience with an observed action, but also the existence of a negative stereotype (see also Cazzato, Makris, & Urgesi, 2017), can influence the level of one’s ability to perceive others’ actions and intentions. However, there is still limited evidence on how explicit and implicit anti-fat attitudes can influence the MR, as well as the underlying neural mechanism.

With regards to the neural underpinnings of MR, a plethora of neurophysiological studies have indicated the existence of an action observation network (AON) involving mainly visual, parietal, and premotor regions (Caspers, Zilles, Laird, & Eickhoff, 2010; Paracampo, Tidoni, Borgomaneri, di Pellegrino, & Avenanti, 2017; Tidoni, Borgomaneri, Di Pellegrino, & Avenanti, 2013). Moreover, the primary motor cortex (M1) has been classically considered to implement a mirror mechanism in perceiving and simulating others’ actions. However, there is so far elusive evidence for whether it is essential for MR (Avenanti, Bolognini, Maravita, & Aglioti, 2007; Naish, Barnes, & Obhi, 2016; Valchev, Tidoni, Hamilton, Gazzola, & Avenanti, 2017). In a recent seminal study, Paracampo, Montemurro, de Vega, and Avenanti (2018) investigated the causal role of M1 in human action prediction by means of transcranial direct current stimulation (tDCS). Their results indicated that tDCS perturbation of the primary motor cortex (M1) could diminish the subjects’ ability to make accurate predictions about the observed actions. This interesting finding provides, for the first time, causal evidence about the role of M1 in the MR mechanism, under diverse methodological parameters (task, tDCS polarity, intensity, and site-specific disruption). However, whether individual differences or the existence of anti-fat attitudes could be linked to a modulation of the MR mechanism under M1 stimulation goes beyond the purpose of Paracampo’s study.

Considering the aforementioned literature, the present study was designed first to replicate and expand our previous behavioural finding (Cazzato & Makris, 2019) that the existence of a weight stereotype can influence the way one perceives and simulates others’ actions. Furthermore, we investigated whether M1 plays a critical role in the simulation of observed actions as part of an extended AON. Indeed, if M1 is essential for action prediction, based on previous findings, we expected that modulating its neural functioning by means of cathodal (disruptive) tDCS also would disrupt performance in the behavioural task. To this aim, we have implemented the same object-weight discrimination task (WDT, see also Finisguerra, Amoruso, Makris, & Urgesi, 2018), in which participants had to indicate whether the observed object (a cube) was light or heavy, along with a crucial manipulation of the weight similarity between the model-actor and the observer-subject. It has been previously shown that bluffing intentions as coded by incongruent kinematics may result in a critical modulation of the MR effect (Finisguerra et al., 2018; Tidoni et al., 2013). As such, our task also involved two different types of action (truthful vs. fake) to gain a better insight on the mechanisms underlying MR. Finally, the task was performed in two counterbalanced sessions performed immediately after active (cathodal tDCS) or sham tDCS over left M1. In keeping with previous studies indicating that cathodal currents over the region reduce M1 excitability (Nitsche et al., 2008; Nitsche & Paulus, 2011; Paracampo et al., 2018; Stagg, Antal, & Nitsche, 2018), we applied a similar protocol for the active tDCS condition (cathodal tDCS, 2mA stimulation intensity, 15-min duration).

Implicit (or automatic) and explicit (self-report) measures of anti-fat attitudes were measured by means of a weight implicit association test (weight-IAT) and the Fat Phobia Scale, respectively. The rationale behind our methodological choice resided in the fact that there are several interesting advantages in investigating both explicit and implicit measures. Particularly, a number of empirical evidence suggests that people often refrain from explicit endorsements of negative attitudes and stereotypes toward social groups (Crosby, Bromley, & Saxe, 1980; Greenwald, Poehlman, Uhlmann, & Banaji, 2009; Teachman & Brownell, 2001). Thus, implicit measures counter some of the limitations of explicit ones, such as response bias and demand characteristics (see Gapinski et al., 2006). Furthermore, research showed that implicit attitudes predict certain forms of behaviour (e.g., nonverbal behaviour and spontaneous behaviour) but not others (see Dovidio, Gaertner, Kawakami, & Hodson, 2002). Hence, assessing both implicit and explicit facets of weight stigma may be deemed necessary to an in-depth understanding of the correlates of anti-fat bias. Furthermore, addressing the unique relationship between people’s ability to predict others’ actions with both implicit and explicit anti-fat attitudes may inform development of interventions for reducing anti-fat bias and for improving the health and well-being of individuals, who are overweight or obese.

In agreement with our previous findings (Cazzato & Makris, 2019), we expected that performance in the WDT, as an implicit measure of MR, would vary as a function of the weight stigma, further corroborated by measures of weight-IAT and Fat phobia scale. More specifically, we expected that at baseline (sham tDCS) both groups of participants would perform better in the task after observing the normal-weight models compared with the overweight ones. Finally, following up from previous evidence with regards to the role of the primary motor cortex in the MR mechanism, we expected that compared with sham tDCS, cathodal tDCS over M1 would disrupt, as implicitly measured by performance in the task, the functionality of M1 and thus highlight the critical role of the primary motor cortex in the simulation and prediction of observed actions.

## Methods

### Participants

A total of 34 subjects (17 normal-weight, 7 females; 17 overweight; 13 females) participated in the study (mean age = 22 years, standard deviation [SD] = 3.4). Participants were recruited internally through the Psychology SONA participation scheme at Liverpool John Moores University. Additionally, participants were recruited externally through poster advertisements situated in public locations, social media, and through individuals known to the researchers. As an incentive, participants either received SONA points and/or £10 in Shopping Vouchers. All subjects were recruited based on their body mass index (BMI). According to the World Health Organisation BMI criteria (WHO, 2000), those with a BMI between 18.5–24.9 were classified as normal-weight (mean = 22.4, SD = 1.5), whereas subjects with a BMI above 25 were classified as overweight (mean = 31.3, SD = 3.9). Participants’ BMI was obtained from measuring weight (Kg) and height (cm), by means of a digital scale (OMRON BF511 full body composition scale) and a stadiometer. All subjects were right-handed, as measured by means of a standard handedness inventory (Briggs & Nebes, 1975; mean = 702.9, SD = 220.5). They reported normal or corrected-to-normal visual acuity, and they were naïve to the purposes of the study. After providing an overview of the experimental procedure, including technical information about tDCS, all subjects provided written, informed concern for participation. Before the tDCS session, all subjects completed a medical screening questionnaire checking for contraindications to the use of tDCS (Rossi, Hallett, Rossini, & Pascual-Leone, 2009). They were all in good health, with no history of neurological or psychiatric disorders and free of psychotropic or vasoactive medication. After the end of the tDCS session, none of the participants complained of any discomfort or adverse effects during the whole procedure. Moreover, at the end of the experiment, participants completed the following self-report questionnaire (1) the Fat Phobia Scale—short form (Bacon, Scheltema, & Robinson, 2001), to measure explicit fat prejudice and (2) The Weight-Implicit Association Test (weight-IAT, Greenwald, Nosek, & Banaji, 2003) to assess implicit anti-fat bias, at the end of which they were debriefed as to the purposes of the study. As expected, an independent sample *t*-test indicated that BMI was significantly higher in overweight than in normal-weight participants. However, the two groups were matched for age, Fat Phobia and Weight-IAT scores (see Table 1). Finally, all the experimental procedures were approved by the Liverpool John Moores University research ethics committee and were in accordance with the ethical standards of the Declaration of Helsinki (1964).

**Table 1:** Mean and standard deviation (SD, in brackets) of participants’ demographics information and scores to the implicit and explicit measures of anti-fat attitudes (weight-IAT and Fat Phobia Scale) as a function of groups’ weight (normal weight vs. overweight)

	Normal weight n = 17	Overweight n = 17	Normal weight vs. overweight
Age	21.29 (3.58)	23.53 (2.92)	*t* (32) = 1.99; *p =* 0.06
BMI	22.43 (1.82)	31.34 (3.95)	*t* (32) = 8.45; *p <* 0.001
Weight-IAT	0.54 (0.28)	0.56 (0.26)	*t* (32) = 0.17; *p* = 0.865
Fat phobia scale	3.56 (0.33)	3.54 (0.52)	*t* (32) = −0.16; *p* = 0.873

Data of participants were compared by means of independent sample *t*-test.

BMI, body mass index; Weight-IAT, weight-implicit association test (D-score).

### General procedure

In two separate days, participants completed the WDT after cathodal or sham tDCS (counterbalanced within subjects and groups of normal weight and overweight participants) delivered over the left M1. Each block lasted for approximately 20 min (tDCS stimulation + task duration). Finally, after the tDCS experiment, participants were required to provide information about their weight and height (for calculating BMI) and to complete the Weight-IAT and the Fat Phobia Scale.

### Weight discrimination task, stimuli, and kinematic analysis

**Fig. 1:**
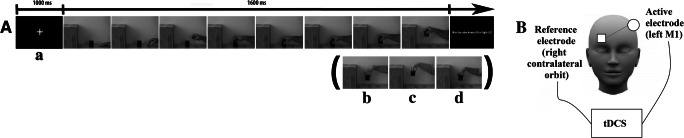
(**A**) Sequence of presentation in a typical trial for the weight discrimination task (adapted from Cazzato & Makris, 2019). Pictures represent video-clip frames during which a) an overweight male model performs a true action, b) a normal weight male model performs a true action, c) an overweight female model performs a true action, and d) a normal-weight female model performs a true action. (**B**) tDCS montage showing the positions of the active and reference electrodes on the left primary motor cortex (M1) and on contralateral orbit, respectively.

The stimuli for the weight discrimination task were the same as in our previous study (see Cazzato & Makris, 2019). They consisted of a series of video clips depicting a normal-weight or overweight male or female model performing a reaching, grasping, and lifting an object action (Fig. 1A). The objects were two metal cubes of the same dimension (5×5×5 cm) and identical appearance, but different weights (“light” approximately 100 g and “heavy” approximately 800 g). All videos were recorded from the posterior plane with a Canon HD Camera, and they were then processed by means of the Adobe Premier Software (Adobe Systems Incorporated, San Jose, CA) in order to appear in black and white, thus controlling for local changes in skin tone and have the same duration of 1,600 ms (split in 8 frames, 200 ms each). During the first part of the recording, the models were informed about the correct weight of the cube. They were instructed to perform a congruent to the object’s weight reaching, grasping, and lifting action (true action [TA]). For the second part, the actors also were informed about the correct weight of the cube. However, this time they were instructed to perform an incongruent action (i.e., to pretend that the cube was light for the heavy one and vice versa for the light one; fake action [FA]). Overall, there were 16 video clips following a 4 actors (2 normal-weight, 2 overweight) × 2 types of action (TA, FA) × 2 metal cubes (light, heavy) design. A subsequent kinematic analysis on the recorded video clips revealed significant differences in the way models handled the different objects for the fake and true trials, but only for the grasping and lifting actions and not the reaching one (for more information on the kinematic analysis procedure and results see Cazzato & Makris, 2019). This is quite important, because we were expecting our subjects to make their judgments about the weight of the depicted object based on the perceived kinematics during the grasping and lifting phases.

During the experiment, all subjects were seated in a dimly lighted testing room in front of a 19-inch LCD monitor (resolution 1,027 × 768 pixels, screen refresh frequency at 60 Hz). The whole experiment was created in and controlled by E-Prime 2.0 Professional Software (Psychology Software Tools, Pittsburgh, PA). At the beginning of the experimental session, subjects were required to provide their demographic information, followed by brief instructions about the task. Participants were reminded to ask any questions about the task. They completed an 8-trial practice block before proceeding with the actual experiment. Each trial started with a centrally located black fixation cross presented on a grey background. After 1 second, the video clips depicting the models performing the true or fake action appeared for 1,600 ms at the centre of the screen subtending a visual angle of approximately 12° × 10°, followed by a question asking the participants to indicate whether the object they saw in the trial was “heavy” or “light.” All 16 video clips were presented in a random order controlled by the E-Prime software. Overall, there were 3 blocks with 32 trials each (16 video clips repeated twice).

### Transcranial Direct Current Stimulation parameters

Transcranial Direct Current Stimulation (tDCS) was delivered using a battery-operated constant direct current stimulator (The Magstim Co. Ltd., Whitland, Carmarthenshire, UK). A pair of surface sponge electrodes (5 × 5 cm) were soaked with a standard saline solution (NaCl .9%) and held in place by elastic rubber bands. To target left M1, the active electrode was placed over the C3 electrode position of the 10-20 system, and the reference electrode was placed on the forehead over the contralateral orbit area (Nitsche & Paulus, 2011) (Figure 1B). Cathodal-tDCS was delivered with a constant current of 2 mA. Stimulation lasted for 15 min, not including 20 seconds of ramp-up and ramp-down at the beginning and end of stimulation, respectively. For sham tDCS, the electrodes were placed on the same locations, but the current was turned on for only 30 seconds at the beginning of the sham session and was then turned off in a ramp-shaped fashion (fade in/out: 20 sec). This way participants experienced the sensations initially associated with the onset of stimulation (mild local tingling), without inducing any effective modulation of cortical excitability. Cathodal and sham tDCS sessions were counterbalanced (for half of the participants the first tDCS session was sham followed by cathodal tDCS on another day, and vice versa for the other half). Neither the subject nor the researcher delivering the stimulation were informed about the type of tDCS (double-blinded). Because this was a within-subjects design, meaning that participants received both sham and cathodal tDCS at different experimental sessions, an interval of at least 48 hours was allowed between the two active and sham stimulation sessions to avoid carryover effects and to guarantee a sufficient washout of the effects of the previous session (Bolognini, Olgiati, Rossetti, & Maravita, 2010; Bolognini, Rossetti, Casati, Mancini, & Vallar, 2011; Mancini, Bolognini, Haggard, & Vallar, 2012).

### Weight-IAT and Fat Phobia Scale

**Fig. 2:**
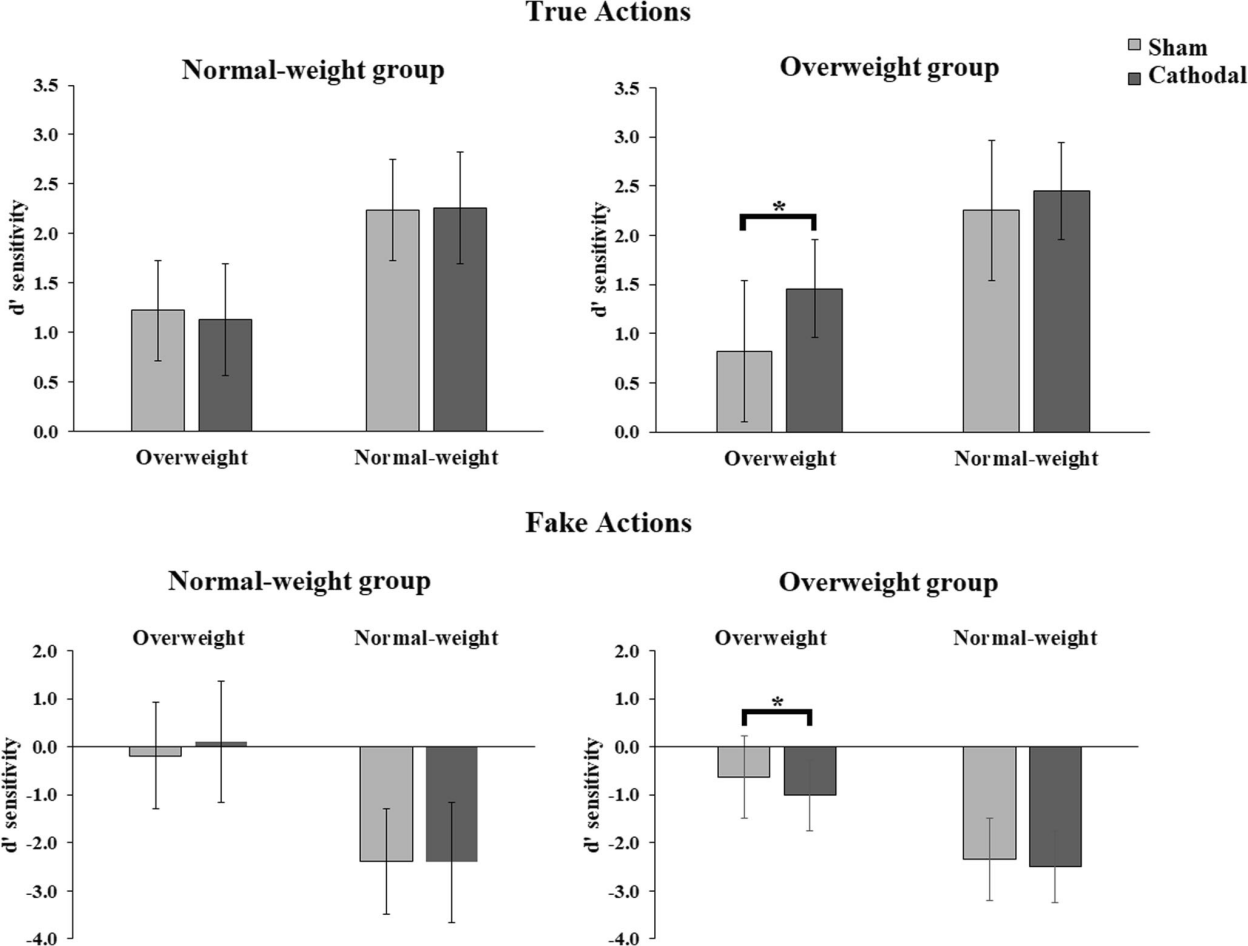
Mean (±SEM) of normal- and overweight models participants’ task sensitivity (*d’*), during observation of true and fake actions performed by normal- and overweight-models upon the light or the heavy object during sham and cathodal tDCS (c-tDCS) over left M1. A higher d’ score corresponds to better task sensitivity. Asterisks indicate significant comparisons (*p* < 0.05).

At the end of the tDCS sessions, all participants completed an implicit, automatic measure of weight bias, i.e., Weight-IAT and one self-report, explicit measure of weight bias, by means of the Fat Phobia Scale. For the Weight-IAT, participants were required to answer as fast and accurately as possible after the onset of the stimuli (i.e., single words or images), by pressing a left (E) or a right (I) key on a computer keyboard with the index finger of the left hand and right hand, respectively. The IAT lasted approximately 8 minutes and was administered in seven blocks, consisting of both congruent and incongruent conditions (blocks 3, 4, 6, and 7) and familiarization blocks (blocks 1, 2, and 5) (Cazzato et al., 2017; Cazzato & Makris, 2019; Greenwald et al., 2003). Before the first presentation of the weight-IAT, participants were shown a list with all the words belonging to the two relevant categories and were asked to read carefully all of the stimuli. In the first block of the weight-IAT, 12 images of overweight and 12 images of normal-weight people were presented and had to be classified as being either “Fat” (left key) or “Normal-weight” (right key) (Cazzato, Siega, & Urgesi, 2012). Each of the 12 images of the two categories was presented only once for a total of 24 trials. The second block also consisted of 24 trials in which negative (requiring a left-key response) and positive (requiring a right-key response) words were presented. Some examples for negative words (belonging to “Bad” category) are “Terrible, Agony, and Horrible.” Some examples for positive words (belonging to “Good” category) are “Joy, Wonderful, and Happy.” In the third block (24 trials practice) and in the fourth block (48 trials test), both overweight and normal-weight bodies and good (positive) and bad (negative) words were randomly presented. Participants were instructed to press the left key for bad-related words and images of overweight people and the right key for good-related words and images of normal-weight people (congruent-stereotype condition). In the fifth block (24 trials), response key assignments were reversed in relation to the categorization involving images of overweight people (right-key) and images of normal-weight people (left-key). Finally, in the sixth block (24 trials practice) and in the seventh block (48 trials test), both overweight and normal-weight bodies and positive and negative words were randomly presented and participants were required to press the left key for images of overweight people and positive words and the right key for images of normal-weight people and negative words (incongruent stereotype condition). Typically, participants are faster and more accurate in the congruent- than in the incongruent-stereotype blocks, thus demonstrating an automatic association between overweight and “Bad” categories and normal-weight and “Good” categories (Greenwald et al., 2003). Stimuli were randomly presented within each block. Each word/image remained on the computer screen until the participant gave a correct response in each trial. Indeed, if an error occurred in a trial, a red “X” appearing below the word stimulus prompted the participant to correct the mistake by pressing the correct key. Following the response, the next stimulus appeared after 500 ms, during which only the category labels were visible on the screen. The response latency data for each participant were transformed into D scores using the D-algorithm, as developed by Greenwald et al. (2003). Accordingly, a positive D score indicates that a participant responded more quickly when categorizing positive adjectives with “Normal-weight” and negative with “Fat,” than when categorizing in the opposite manner (“Normal-weight” with negative and “Fat” with positive) Fig. 2.

Finally, all participants filled the Fat Phobia Scale-short form self-report questionnaire (Bacon et al., 2001), which assessed explicit negative attitudes and stereotyped perceptions of obese people. In this measure, 14 pairs of adjectives are used to describe obese people (e.g., “lazy” vs. “industrious”; “no will power” vs. “has will power”), and respondents are asked to indicate on a scale from 1 to 5 which adjective they feel best describes their beliefs about obese people. A score of 2.5 indicates neutral attitudes about obese persons. Scores more than 2.5 reflecting higher levels of fat phobia (more negative attitudes) and lower scores indicating more positive attitudes.

### Data handling

Behavioural performance obtained at the two-alternative forced-choice WDT after sham and cathodal tDCS sessions was analysed using the signal detection theory (SDT). Based on SDT, we calculated d′ as a measure of (perceptual) sensitivity and lnβ as a measure for the response bias. The percentage of correct responses (accuracy) was first calculated for each participant in each experimental condition**.** In keeping with our previous study (Cazzato & Makris, 2019), the SDT parameters (d′ and lnβ scores) were calculated, considering whether subjects’ responses were congruent or not to the real weight of the cube. This way “heavy-object” responses to heavy-object stimuli were considered as hits and “heavy-object” responses to light-object stimuli as false alarms. D′ and lnβ scores data from the WDT were then entered into a mixed four-way 2 × 2 × 2 × 2 ANOVA with: 2 (tDCS stimulations: cathodal tDCS, sham tDCS) × 2 (model’s weight: normal-weight, overweight) × 2 (type of action: true, fake) as within-subject factors and the subject’s weight (normal-weight, overweight) as a between-subject factor. Finally, we calculated, for each condition, a measure of the change of measure of perceptual sensitivity d’ scores as the ratio between the individual values after cathodal tDCS and the corresponding values in the sham tDCS condition [cathodal tDCS/sham tDCS]. The change indices were correlated, using Pearson correlations, with individual scores obtained at the Fat Phobia Scale and the Weight-IAT. All statistical analyses were performed with STATISTICA 8.0 (StatSoft Inc, Tulsa, Oklahoma). The source of all significant repeated-measure ANOVA interactions was analysed using the Duncan’s post-hoc tests. Effect sizes were estimated using the partial eta square variable (ηp2). All data are reported as mean (M) and standard error of the mean (SEM). A significance threshold of *p* < 0.05 was set for all effects.

## Results

### Implicit and explicit anti-fat attitudes

Further to the Weight-IAT, one-sample *t* tests were used to compare the mean D-scores to zero (where zero refers to the absence of any response bias) for both groups. Both normal-weight and overweight participants showed a significant stereotypical anti-fat bias, indicating that they were more prone to associate overweight people to the bad-related category and normal-weight people to the good-related category than vice versa [normal-weight group: t(16) = 7.89, *p* < 0.001; overweight group: t(16) = 8.7, *p* < 0.001]. Interestingly, the two groups did not differ in the levels of stereotypical explicit fat phobia. One-sample *t* tests also were performed to compare the mean Fat phobia scale scores to 2.5 (where 2.5 refers to a moderate level of explicit phobia against obese people) for each group. In accordance with implicit anti-fat bias results, both normal-weight and overweight participants showed high level of explicit negative attitudes and stereotyped perceptions of obese people (normal-weight group: t(16) = 13.46, *p* < 0.001; overweight group: t(16) = 8.24, *p* < 0.001).

### WDT performance after sham and cathodal tDCS

The 2 × 2 × 2 × 2 ANOVA on the mean d′ scores revealed main effects of subject’s weight [*F*(1,32) = 8.041, *p* < 0.001, *ηp*^*2*^ = 0.201], model’s weight [*F*(1,32) = 31.510, *p* < 0.001, *ηp*^*2*^ = 0.496] and of action type [*F*(1,32) = 58.598, *p* < 0.001, *ηp*^*2*^ = 0.647], further corroborated by a significant two-way interaction between model’s weight and action type [*F*(1,32) = 123.547, *p* < 0.001, *ηp*^*2*^ = 0.794], as well as a significant two-way interaction of model’s weight × subject’s weight [*F*(1,32) = 9.352, *p* < 0.001, *ηp*^*2*^ = 0.226]. Most importantly, the four-way interaction of tDCS stimulations, model’s weight, action type, and subject’s weight was also significant [*F* (1,32) = 5.655, *p* = 0.024, *ηp*^*2*^ = 0.150].

For truthful actions trials, post-hoc comparisons showed that compared to sham stimulation, cathodal tDCS over M1 yielded no effects while normal-weight participants were observing either overweight (sham tDCS: 1.22 ± 0.34 vs. cathodal tDCS: 1.13 ± 0.28, *p* = 0.564) or normal-weight models (sham tDCS: 2.24 ± 0.37 vs. cathodal tDCS: 2.26 ± 0.34, *p* = 0.902) performing a true action.

On the opposite, cathodal tDCS over M1 improved perceptual sensitivity when overweight participants observed overweight models performing true actions (sham tDCS: 0.82 ± 0.34 vs. cathodal tDCS: 1.45 ± 0.28, *p* = 0.001). This effect was not detected when overweight participants were observing normal-weight models performing a true action (sham tDCS: 2.25 ± 0.37 vs. cathodal tDCS: 2.45 ± 0.34, *p* = 0.258).

For fake actions trials, compared with sham stimulation, cathodal tDCS over M1 did not affect normal-weight participants’ sensitivity scores while they were observing either overweight (sham tDCS: −0.18 ± 0.35 vs. cathodal tDCS: 0.11 ± 0.32, *p* = 0.08) or normal-weight models (sham tDCS: −2.39 ± 0.36 vs. cathodal tDCS: −2.41 ± 0.33, *p* = 0.902) performing a fake action.

On the opposite, cathodal tDCS over M1 decreased the perceptual sensitivity whilst overweight participants were observing an overweight model performing fake actions (sham tDCS: 0.82 ± 0.34 vs. cathodal tDCS: 1.45 ± 0.28, *p* = 0.026). This effect was not evident when overweight participants were observing normal-weight models performing a fake action (sham tDCS: −2.34 ± 0.36 vs. cathodal tDCS: −2.50 ± 0.33, *p* = 0.379).

To sum up, cathodal tDCS over M1 improved the ability of overweight participants to simulate the true actions when performed by the overweight models and thus to make accurate responses in the task. Furthermore, cathodal tDCS improved their action simulation when observing fake actions performed by overweight models, because they were more fooled by their deceptive kinematics, and thus, they made more error responses in the task. Finally, for lnβ scores the ANOVA revealed no significant main effects or interactions (all Fs < 3.8; *p* > 0.05), confirming that the aforementioned effects were not mediated by a change in response bias.

Finally, no significant correlations were found between the tDCS indices and implicit weight bias (weight-IAT) or explicit fat phobic attitudes (Fat Phobia scale) scores for any of the models or group of participants.

## Discussion

In the present study, we investigated how inhibitory versus control noninvasive brain stimulation over the left primary motor cortex (M1) can modulate the simulation and understanding of observed familiar actions in terms of a weight discrimination task. More specifically, we applied cathodal (inhibitory) and sham (control) tDCS over the M1 area of normal-weight and overweight participants, prior to observing normal-weight and overweight models performing truthful or fake reaching and grasping actions and making a decision about the weight of the object. Furthermore, at the end of the WDT, the two groups were required to complete the Weight-IAT and the Fat Phobic scale to investigate associations between explicit and implicit weight stereotypes and the effects obtained on the WDT after cathodal tDCS stimulation.

In keeping with our previous (behavioural) study (Cazzato & Makris, 2019), the statistical analysis of the WDT has revealed that during sham tDCS, both normal-weight and overweight participants performed better when they observed the normal-weight models performing the actions compared with the overweight ones. Most importantly, tDCS over M1 has, surprisingly, improved the way that our overweight participants were able to perceive and simulate the actions performed by the overweight models. No such finding was observed in the normal-weight group or for the normal-weight models. More specifically, for true actions, cathodal tDCS over M1 resulted in better performance in the WDT when overweight participants observed the actions performed by the overweight models, indicating that the inhibition of M1 resulted in an increase of the level at which the participants could simulate the observed actions and thus make accurate judgments about the weight of the object. The opposite pattern was observed for fake actions; i.e., their performance in the task was worse as compared to sham stimulation, thus indicating that they were better into simulating the deceptive kinematics, being fooled, and making an inaccurate response. Finally, even though both groups of normal-weight and overweight participants displayed similar and higher level of explicit fat phobia and negative attitudes towards obese people, we did not find correlational evidence to suggest an association between explicit and implicit weight stereotypes and the effects obtained on the WDT after active tDCS stimulation. Despite these unexpected effects, the aforementioned findings provide some further evidence on the role of M1 in action observation and MR.

Earlier studies on the neural basis of the motor resonance mechanism have described the existence of an action observation network (AON) responsible for the simulation of observed action sequences and the prediction of their outcome (Springer, Parkinson, & Prinz, 2013; Urgesi et al., 2010). Initially, in those studies the AON comprised mainly of visual, parietal, and premotor areas, whereas M1 was not considered part of the network, as previous neurophysiological and neuroimaging studies did not reveal a consistent activation of M1 during action observation (Caspers et al., 2010; Gazzola & Keysers, 2009). More recently, however, a series of neurophysiological studies has revealed a modulation of neuronal activity in M1 during action observation, similar to that of action execution, thus leading researchers to propose that M1 could be considered part of an extended AON (Naish, Houston-Price, Bremner, & Holmes, 2014; Valchev et al., 2015; Vigneswaran, Philipp, Lemon, & Kraskov, 2013). In a more recent study, Paracampo et al. (2018) have shown that cathodal tDCS over M1 impaired the participants’ accuracy in an action prediction task, but only when the observed actions were performed by humans compared with nonhumans. Thus, they provided seminal causal evidence of the critical role of M1 in action simulation and prediction.

In the present study, we applied a tDCS protocol similar to that described by Paracampo et al. (2018). Our findings are somewhat comparable to those reported in their paper, in the sense that cathodal stimulation over M1, compared with sham, has indeed modulated the way that our overweight participants perceived and simulated the viewed actions. Moreover, our WDT was similar to their action prediction task, in the sense that in both studies subjects had to perceive accurately the kinematics of the models to make accurate predictions for the weight of the object (present study) or the size of the object (Paracampo et al., 2018). A critical difference was that our methodological manipulation allowed for the investigation of whether the existence of negative attitudes towards overweight models can modulate performance in the task as a function of the MR mechanism. Indeed, despite the lack of correlational evidence, both groups strongly endorsed negative stereotypes against fat people, indicating that anti-fat bias can modulate the level of simulating and understanding familiar actions performed by normal-weight or overweight people and that this effect on its own may be altered by the type of stimulation we applied over M1. Hence, Paracampo et al. (2018) were the first to provide causative evidence on the critical role of M1 in the MR mechanism. However, the findings of the present study have expanded on these results, by providing further insight on how top-down influences, such as the existence of anti-fat attitudes, can affect the MR mechanism and the involvement of M1 in understanding others’ actions.

One consideration here is that cathodal tDCS only in our overweight subjects modulated the simulation of fake or true actions performed by the overweight models. No such modulation was observed in our slim subjects or for actions performed by the normal-weight models. One could argue that if M1 is part of the AON and thus, involved in action perception and prediction, tDCS should modulate the measured effects in the task, irrespective of the physical characteristics of the actors, similar to the study of Paracampo et al. (2018). This is indeed a surprising finding and one explanation we could provide for that is the fact that in the current study a critical methodological manipulation was the effect of the anti-fat attitudes in simulating the actions of overweight people. In line with previous findings (Cazzato & Makris, 2019), we have shown that during sham tDCS, both normal-weight and overweight subjects endorsing high levels of explicit fat phobia measures are worse at detecting and simulating the action kinematics of overweight actors, as opposed to normal-weight ones. Hence, it could be that the simulation of the actions performed by the normal-weight models had reached ceiling levels that could not be further modulated by the tDCS.

Moreover, an alternative, but not mutually exclusive explanation to this could reside in other individual differences (which were not accounted for in this study), for example in the emphatic responses of our participants to normal-weight compared with overweight individuals. With these regards, there is increasing research evidence of an association between the magnitude of cortical activity in the AON and the primary motor cortex and empathic responses to others (Avenanti, Bueti, Galati, & Aglioti, 2005; Gazzola, Aziz-Zadeh, & Keysers, 2006; Perry, Troje, & Bentin, 2010; Zaki, Weber, Bolger, & Ochsner, 2009). Recently, a study by Jospe, Flöel, and Lavidor (2020) indicated that manipulating levels of motor excitability, by means of noninvasive brain stimulation, can modulate the level of understanding and simulating others’ actions. More specifically, they have shown that tDCS interference over M1 has impaired or improved their subjects’ ability to perceive and simulate hand gestures, depending on their levels of empathy. Even though in the present study we have not measured or controlled for levels of empathy, it would not be surprising if both our normal-weight and overweight participants, due to their increased levels of anti-fat attitudes, would show less empathic responses for the overweight models (see also Lewis & Hodges, 2011). Therefore, according to the aforementioned literature, it is possible that tDCS over M1 could have improved the empathic resonance of our overweight subjects towards the actions of the overweight models (in group), thus improving their ability to simulate their action kinematics. Nevertheless, we argue that this is an assumption we have not tested here, and further research is deemed as necessary to validate this hypothesis.

Furthermore, there is increasing research evidence showing that M1 possesses functional and reciprocal connections with the somatosensory cortex (Bonini, 2017; Gazzola & Keysers, 2009) and that SI is involved in perceiving proprioceptive information from observed actions (Bolognini et al., 2011; Holle, Banissy, & Ward, 2013; Keysers et al., 2004). More recently, Valchev et al. (2017) have shown that offline cTBS over SI, but not M1, impaired the subjects’ ability to accurately detect the weight of an object lifted by an actor. They concluded that while SI is mainly involved in action perception by extracting proprioceptive/tactile information derived from observed action kinematics, the role of M1 in action simulation is rather debatable and many consider its activity to be a simple downstream consequence of the reciprocal cortico-cortical connections with SI (Geyer, Schormann, Mohlberg, & Zilles, 2000; Rizzolatti & Luppino, 2001). If this is the case, we hypothesize that cathodal tDCS disruption of M1 may have reduced any noise in the network that allowed our overweight participants to better simulate the observed actions from those that they share the same or similar proprioceptive input, i.e., the overweight models. We assume that the same effect was not observed in the normal-weight subjects, as their performance in the task had already reached ceiling effects, irrespective of the type of stimulation. Moreover, Valchev et al. (2017) reported that disruption of M1 had variable effects across their participants, as in half of their subjects it increased their performance to the task. A similar finding has been also reported by Palmer, Bunday, Davare, and Kilner (2016) where cTBS over M1 had variable effects across their participants when they were observing familiar actions, leading to inhibition of M1 excitability in some and increases in others. Nevertheless, the aforementioned studies suggest that the observation of both implied and real action sequences can modulate sensorimotor integration (Concerto et al., 2015) and even though in the present study we have not tested for that, we cannot rule out that in some extent our findings could be the outcome of a contribution of brain areas functionally connected to M1 (Mineo et al., 2018).

Finally, it should be noted that our main statistical approach in the present study for analysing the subjects’ performance in the task was a measure of sensitivity into detecting the appropriate kinematics, as opposed to measures of response accuracy. More specifically, instead of just measuring how well the subjects performed the task (response accuracy), by applying the signal detection theory we investigated the level at which they were able to perceive differences in the observed kinematics, which was critical for making accurate judgments in the task, but also for revealing the involvement of the MR mechanism. Thus, we speculate that inhibitory tDCS over M1 could have altered the level of sensitivity for detecting accurate (true actions) or incongruent (fake actions) kinematics, but only for the models that the subjects trusted less, i.e., the overweight ones. Indeed, there is some research literature showing that the anti-fat bias can make overweight people appear less trustworthy (Vartanian, Pinkus, & Smyth, 2014) and that could affect the level at which people perceive and simulate their actions. If our hypothesis is correct, then tDCS interference over M1 could have altered the way participants approached and perceived the actions performed by the overweight models, thus the increased sensitivity we have detected in our results.

Some limitations should be considered, though, when interpreting the findings from this study. The first one has to do with methodological limitation due to the low spatial resolution of tDCS, as well as the montage we have applied for the reference electrode. Even though traditionally tDCS has less spatial resolution compared with other brain stimulation techniques (i.e., transcranial magnetic stimulation, TMS), increasing research literature shows that tDCS stimulation over M1 can successfully interfere with the area’s involvement in action perception and execution (Nitsche & Paulus, 2000, 2001). Nevertheless, some recent studies have indicated that response to tDCS stimulation can be quite variable between participants (Nitsche & Paulus, 2001; Tremblay, Beaulé, Lepage, & Théoret, 2013), with some of them reporting that only 60% of subjects are experiencing the classic tDCS interference effect, thus supporting the need for a method of individualizing tDCS dosage which uses electric-field (E-field) modelling (Bikson, Rahman, & Datta, 2012; Datta, Truong, Minhas, Parra, & Bikson, 2012; Evans et al., 2020), to allow for more consistent responses to tDCS stimulation (Caulfield et al., 2020). We acknowledge that in the present study we did not test our participants for a canonical response to M1 tDCS before enrolling them. We agree that future tDCS studies should cautiously account for this limitation. With regards to the montage of the reference electrode over the right supraorbital area, it is thought that extracephalic electrode montages allow more focal stimulation (Cogiamanian, Marceglia, Ardolino, Barbieri, & Priori, 2007) and so that the location of the reference electrode may have induced a spread of the cathodal current in anterior parts of the brain that could have contaminated our findings (Datta et al., 2009; Im, Park, Shim, Chang, & Kim, 2012). However, whilst a previous study from Avenanti, Paracampo, Annella, Tidoni, and Aglioti (2018) provided the first causal evidence that the Inferior Frontal Cortex is involved not only in planning the execution of an upcoming action, but also in making predictions about the outcomes of observed actions, to the best of our knowledge, most indications point to the involvement of motor and premotor cortexes in the understanding of other people actions (Avenanti et al., 2005; Gazzola et al., 2006; Zaki et al., 2009) and not to the anterior prefrontal cortex, whereas the fact that the current return from the electrode to the cortex dispersed over a large area, would make it less likely to produce significant effects (see also Jospe et al., 2020).

## Conclusions

In the present study, we addressed the question of whether the anti-fat bias can affect the way that we perceive and simulate the actions of other people, in line with the theory of motor resonance. Moreover, we investigated how inhibitory non-invasive brain stimulation over the primary motor cortex can modulate these effects. We have shown that inhibitory tDCS over M1 can modulate motor resonance effects, thus providing further evidence on the role of M1 in action perception and prediction. However, further studies on this topic are deemed necessary to validate, clarify, and further expand those findings.
